# Predicting University Adjustment from Coping-Styles, Self-Esteem, Self-Efficacy, and Personality: Findings from a Survey in a Sample of Italian Students

**DOI:** 10.3390/ejihpe11030066

**Published:** 2021-08-17

**Authors:** Giusy Danila Valenti, Palmira Faraci

**Affiliations:** Faculty of Human and Social Sciences, University of Enna “Kore”, 94100 Enna, Italy; palmira.faraci@unikore.it

**Keywords:** university adjustment, freshmen, coping, self-esteem, self-efficacy, personality

## Abstract

Starting university life requires that students learn to cope with several personal, academic, and social challenges. A wide array of variables affects how students adjust to university life. This study was aimed to investigate which factors among coping styles, self-esteem, self-efficacy, and personality traits (i.e., diligence, relational availability, mental flexibility, activity, and emotional stability) best predicted the levels of university adjustment in a sample of university freshmen (*N* = 204, 63% women). Data were collected using self-report instruments. Multiple regressions analyses were conducted to identify the most significant predictors of adjustment to college. Our findings reported that self-efficacy, task-, and emotion-oriented coping were the most significant predictors, together with relational availability and mental flexibility. These findings might improve the growing knowledge concerning university adjustment, supporting main previous research. The observed relationships between university adjustment and the measured variables suggest intriguing considerations about the importance for schools and universities of providing interventions for students that aim to develop and strengthen the investigated personality facets, reducing withdrawal, behavioral and/or mental disengagement, and promoting academic achievement and success.

## 1. Introduction

The shift from high school to university represents an important life phase encountered by many young adults who have to take on numerous personal and academic challenges. Indeed, the university environment provides new opportunities for students to grow up intellectually, acquire new skills, and learn how to become more independent [1]. On the other hand, students may encounter different stressors, such as the creation of new social bonds with colleagues and teachers—which are usually characterized by a greater level of formality—and the modification of existing relationships with parents, as starting university life often coincides with their first experience far away from the family unit [2,3]. In addition, university life requires that students learn to cope with several tasks, run into new academic demands, take responsibility in their personal lives, and make career choices [4,5,6].

Some freshmen are able to deal with these transitional challenges and adjust to university life successfully, whereas others feel overwhelmed and experience various adjustment problems leading them to drop out of university [7,8]. Adjustment to college has been considered as an important factor in predicting university outcomes in terms of grades, retention, withdrawal, or success [9,10]. Other possible consequences concern students’ psychological symptoms, such as depression, loneliness, homesickness, and sadness [8,11,12], when adjustment to university life is lacking or poor.

Despite the complex nature of the college environment, there is substantial agreement about the structure of the broad university adjustment construct. A significant conceptualization of university adjustment derives from the taxonomy proposed by Baker and Syrik [13], which is well accepted and also widely used in more recent studies. It distinguishes four different domains, namely academic adjustment, social adjustment, psychological adjustment, and institutional attachment. Academic adjustment refers to students’ ability in coping with the academic demands, such as the amount of study or the attendance at courses; social adjustment concerns the quality of the social networks with teachers and colleagues and being involved in social activities; psychological adjustment reflects students’ general wellbeing, the degree of stress and anxiety, and/or the physical reactions to the demands of the college environment; institutional attachment refers to students’ feelings of commitment and satisfaction with attending a specific university and the degree of emotional attachment to the university community. 

As suggested by the literature on university adjustment, a large number of factors may affect how students adjust to university life. For instance, coping styles, self-esteem, self-efficacy, and personality traits are among the most investigated variables that are related to university adjustment. Understanding the influence of these variables on college students’ adaptation may be useful for parents, teachers, and educators to help them to better experience this transition.

### 1.1. Coping Styles and University Adjustment

According to Lazarus and Folkman [14], coping responses are the cognitive and behavioral attempts and efforts that individuals use in order to handle, tolerate, or reduce the effects of stressful life events. Coping styles have been dichotomized into problem- and emotion-focused coping strategies. The former have been considered functioning and adaptive, the latter have been associated with poor or maladaptive outcomes [15]. Further descriptions of coping styles have identified a third type, named avoidance-focused coping strategies, also associated with low levels of adjustment [16].

From the meta-analytic findings by Credé and Niehoster [4], associations with coping styles have come to light, supporting the well shared view regarding the link between problem-focused coping strategies and adjustment, on the one hand, and emotion- and avoidance-focused coping strategies and adjustment difficulties on the other, and similar results were also found in further studies [3,12,17,18,19]. 

### 1.2. Self-Esteem and University Adjustment

Self-esteem concerns global feelings of self-worth, or a generalized feeling of self-acceptance, goodness, and self-respect [20], and it is often associated with several positive outcomes. Empirical findings about the associations between self-esteem and university adjustment appear incongruent and inconsistent. A possible explanation of these incoherent results may depend on how self-esteem is conceptualized, that is, if it is assessed as a global construct or, otherwise, if particular facets are taken into account [21]. When self-esteem is intended as a global concept, significant and positive associations with university adjustment are generally estimated [22,23,24], whereas when multiple subdimensions are assessed, the links to university adjustment may show different patterns. For instance, Pasha and Munaf [25], reported that some components of self-esteem (i.e., competence, lovability, personal power, moral self-approval, body functioning, and likeability) were significantly and positively related to university adjustment, whereas other components (i.e., self-control and identity integration) did not show any significant associations. These findings emphasize how difficult the analysis of the contribution of self-esteem in predicting university adjustment is.

### 1.3. Self-Efficacy and University Adjustment

Self-efficacy is described as “the belief in one’s capabilities to organize and execute courses of action required to produce given attainments” [26] (p. 3). It refers to the individual’s skills to perform appropriately in challenging situations.

Self-efficacy seems to strongly influence university adjustment [26,27], as individuals with a high sense of self-efficacy tend to feel able to overcome situational difficulties, giving to them a low stressful meaning. Some findings supported the relationship between the two constructs, showing significant positive associations [4,28].

### 1.4. Personality Traits and University Adjustment

The Big-Five trait model of personality represents the most widely accepted and commonly used conceptualization of personality. A plethora of studies have been conducted to examine the extent to which each trait affects individuals’ adaptation and wellbeing, and results of literature seem to converge in supporting that all traits, to a various degree, have positive effects, whereas neuroticism is linked to negative outcomes [29]. 

In line with these premises, findings from the meta-analytic review by Credé and Niehorster [4] revealed that all personality traits had positive and moderate relationships with university adjustment, except for neuroticism, which reported inverse associations of the same strength. Similar results were also found in further studies [30,31,32]. Nevertheless, it is worth noting that some authors argued that studies using the Five-Factors model conceptualize personality traits broadly limiting the specificity of their predictions on adjustment to college, suggesting that a more detailed description of personality—such as Cattell’s taxonomy—might provide a more comprehensive view regarding the associations between personality traits and the outcome variable [33].

### 1.5. The Current Study

To the best of our knowledge, the associations between university adjustment and a large set of variables has not been assessed in a unique study sample. In addition, some of the previous findings did not examine all the domains of the multidimensional university adjustment construct, analyzing, indeed, one or two aspects of it. The purpose of this study was to examine which factors among coping styles, self-esteem, self-efficacy, and personality traits best predicted the levels of university adjustment among university freshmen. 

Based on previous empirical findings, the current study was aimed at testing the following hypotheses:

**Hypothesis** **1** **(H1).** *Problem-focused coping strategies will be positively related to university adjustment, whereas emotion- and avoidance-focused coping strategies will show associations of the opposite direction.*

**Hypothesis** **2** **(H2).** *Self-esteem will be positively related to university adjustment.*

**Hypothesis** **3** **(H3).** *Self-efficacy will be positively related to university adjustment.*

**Hypothesis** **4** **(H4).** *Personality traits will be positively related to university adjustment, except for neuroticism, which will report associations of the opposite direction.*

## 2. Materials and Methods

### 2.1. Participants

Participants were 204 college students, 129 women (63%) and 75 men (36.8%), with a mean age of 20.65 (*SD* = 1.63), enrolled at University Kore of Enna, Italy, a no-state mid-sized university which encompasses the following degree courses: Architecture, Economics, Engineering, Foreign Languages, Law, Literature Sciences, Medicine and Surgery, Primary Education Sciences, Psychology, Social Services and Forensic Sciences, Sport Sciences, and Strategic and Safety Sciences. Before filling out the survey created for this study, students were asked to specify which department they belonged to. Table 1 displays the distribution of participants by university departments. An a priori power analysis using G*Power [34] was performed to estimate the minimum recommended sample size for multiple regression analyses. With a medium effect size (f^2^ = 0.15), α = 0.05, power of 0.95, and 10 predictors, the required sample size was *N* = 172. Therefore, the number of participants involved in our study was adequate for the following analyses.

### 2.2. Procedure 

Data were gathered in the public spaces of the university campus (e.g., libraries, study rooms, and canteens) by informal contacts with the first author. The instruments were presented in a random order to avoid possible biases related to tiredness or decrease in attention. Data collection was conducted at the end of the second semester, and students were selected if they were attending their first academic year. Being a first-year college student was the unique criterion for participants’ selection. Individuals were informed that their participation in the study was voluntary, and they were also assured of the confidentiality of the information obtained. Informed consent was obtained by all participants prior to answering the survey. The study was approved by the Internal Review Board of Research in Psychology of UKE2.3.

### 2.3. Measures

- Student Adaptation to College Questionnaire (SACQ) [35]

The Student Adaptation to College Questionnaire was used to assess adjustment to university life. It is a 67-item questionnaire, with a 9-point Likert scale (from 1 “Does not apply to me at all” to 9 “Applies very closely to me”), with higher scores indicating higher levels of adjustment to college. The SACQ includes four scales: SACQ Academic Adjustment (α = 0.90), SACQ Social Adjustment (α = 0.86), SACQ Psychological Adjustment (α = 0.84), and SACQ Institutional Attachment (α = 0.86).

The academic scale refers to students’ ability in coping with the educational demands; the social scale concerns students’ ability in facing the interpersonal and social requests of college life; the psychological scale measures students’ general psychological wellbeing; and the institutional attachment refers to students’ feelings of commitment and satisfaction with attending a specific university, and the degree of emotional attachment to the university community. We first translated the original items into the Italian language and then, through a back translation procedure, we translated items again into the original language.

- College Adaptation Questionnaire (CAQ) [36]

The College Adaptation Questionnaire was used to assess participants’ adjustment to university life. It is a unidimensional scale consisting of 18 items, on a Likert-scale (from 1 “Strongly disagree” to 7 “Strongly agree”), aimed to evaluate global and general level of university adjustment. High scores reflect high levels of university adjustment. The reliability coefficient of the scale in this study was 0.87. The forward-backward translation was also applied in this case.

- General Self-Efficacy (GSE) [37]

The General Self-Efficacy scale is a unidimensional measure assessing general self-efficacy. It consists of 17 items and uses a 5-point Likert scale (from 1 “Strongly Disagree” to 5 “Strongly Agree”). In the current sample, Cronbach’s alpha was 0.90.

- Coping Inventory for Stressful Situations (CISS) [38,39]

The Coping Inventory for Stressful Situation is a 48-item questionnaire, with a 5-point Likert scale (from 1 “Not at all” to 5 “Very much”), assessing coping styles. It is a multidimensional measure including three scales: Task, Emotion, and Avoidance. The task-oriented coping scale describes task-oriented efforts aimed at solving a problem; the emotion-oriented coping scale reflects activities meant to reduce negative emotional reactions caused by a problem; the avoidance-oriented coping scale refers to all cognitive changes or attempts to avoid a stressful situation. All of the subscales reached a good internal consistency, with Cronbach’s alphas ranging from 0.85 to 0.88.

- Self-Report Questionnaire [40]

The Self-report Questionnaire is composed of 84 items assessing self-esteem and personality traits, based on the Big-Five model. These personality traits refer to the well-known conscientiousness, agreeableness, openness, extraversion, and neuroticism, though they are renamed, respectively, diligence (α = 0.79), relational availability (α = 0.65), mental flexibility (α = 0.59), activity (α = 0.75), and emotional stability (α = 0.78). A sixth scale is added to assess self-esteem (α = 0.85), and a control scale (α = 0.70) is devoted to examine the possible presence of falsified profiles. The Self-Report Questionnaire uses a 4 point- Likert scale (from 1 “Certainly false for me” to 4 “Certainly true for me”). 

### 2.4. Data Analyses

Descriptive statistics were conducted for each administered instrument to examine the levels of the observed variables. A series of multiple regression analyses were performed to evaluate how well coping styles, self-esteem, self-efficacy, and personality traits predicted the levels of adjustment to college. Five separated multiple regression analyses were conducted, considering both general and specific domains of university adjustment. Data analyses were performed by using IBM SPSS (version 20). 

## 3. Results

### 3.1. Descriptives and Correlations between the Study Variables

Descriptives for the measured variables are presented in Table 2. Both SACQ and CAQ scores reflect a quite satisfactory level of adjustment to college in all domains, and a similar pattern concerns all of the other variables. Furthermore, the control scale scores could be considered as a proof reducing the likelihood of the presence of falsified profiles. In addition, values of skewness and kurtosis do not exceed |1|, showing that all the variables are normally distributed. In Table 3, the correlations between the study variables are shown, and they reported significant results (0.141 < *r < 0*.794). The absence of asymmetry and multicollinearity provide support for the appropriateness of performing regression analyses.

### 3.2. Regression Analyses

#### 3.2.1. Predicting SACQ Social Adjustment

Table 4 shows the results of the first simultaneous multiple regression analysis, examining the effects of the aforementioned independent variables on SACQ Social Adjustment. When these predictors were entered into the regression model, *R* was significantly different from zero, *F* (10, 193) = 14.39, *p* < 0.001. Together, the independent variables accounted for 43% of the variance of the social domain of university adjustment, _Adj_*R*^2^ = 0.39. This regression analysis established that self-efficacy was the best predictor of SACQ Social Adjustment, β = 0.40, *t* (193) = 4.13, *p* < 0.001, followed by CISS Task, β = 0.20, *t* (193) = 2.70, *p* < 0.01, CISS Emotion, β = −0.19, *t* (193) = −2.51, *p* < 0.05, relational availability, β = 0.16, *t* (193) = 2.47, *p* < 0.05, and mental flexibility, β = 0.15, *t* (193) = 2.01, *p* < 0.05. The squared semi partial correlation coefficients indicated that the unique association between self-efficacy, CISS Task, CISS Emotion, relational availability, mental flexibility, and SACQ Social Adjustment were, respectively, 5.06% (*r*_sp_ = 0.23), 2.16% (*r*_sp_ = 0.15), 1.88% (*r*_sp_ = 0.057), 1.82% (*r*_sp_ = −0.14), and 1.19% (*r*_sp_ = 0.11).

#### 3.2.2. Predicting SACQ Academic Adjustment

The results of the second simultaneous multiple regression analysis, assessing the contributions of the independent variables to SACQ Academic Adjustment, are also significant, *F* (10, 193) = 20.53, *p* < 0.001, *R*^2^= 0.52, _Adj_*R*^2^ = 0.49, and they are described in Table 5. The significant predictors were found to be CISS Task, β = 0.30, *t* (193) = 4.36, *p* < 0.001, CISS Emotion, β = −0.30, *t* (193) = −4.17, *p* < 0.001, and self-efficacy, β = 0.21, *t* (193) = 2.36, *p* < 0.05. The unique association between CISS Task and SACQ Academic Adjustment was 4.8% (*r*_sp_ = 0.22), between CISS Emotion and SACQ Academic Adjustment was 4.4% (*r*_sp_ = 0.21), whereas the unique association with self-efficacy was estimated 1.4% (*r*_sp_ = 0.12).

#### 3.2.3. Predicting SACQ Psychological Adjustment

Table 6 shows the results of the third simultaneous multiple regression analysis, which examined the contributions of the independent variables to SACQ Psychological Adjustment. The regression model had a satisfactory fit, *F* (10, 193) = 11.62, *p* < 0.001, *R*^2^ = 0.38, _Adj_*R*^2^ = 0.34, and CISS Emotion was the only significant predictor, β = −0.50, *t* (193) = 6.27, *p* < 0.001. The squared semipartial correlation coefficient indicated that the unique association between CISS Emotion and SACQ Psychological Adjustment was 13% (*r*_sp_ = 0.36).

#### 3.2.4. Predicting SACQ Institutional Attachment

The fourth simultaneous multiple regression analysis assessed how well the independent variables predicted SACQ Institutional Attachment, whose results are described in Table 7. Overall, they accounted for 43% of the variance of SACQ Institutional Attachment, _Adj_*R*^2^ =0.40. The significant predictors were found to be CISS Task, β = 0.29, *t* (193) = 3.73, *p* < 0.001, CISS Emotion, β = −0.27, *t* (193) = −3.51, *p* < 0.001, self-efficacy, β = 0.31, *t* (193) = 3.27, *p* < 0.001, and relational availability, β = 0.23, *t* (193) = 3.49, *p* < 0.001.

The unique association between CISS Task and SACQ Institutional Attachment was 4% (*r*_sp_ = 0.20), between both CISS Emotion and SACQ Institutional Attachment and between relational availability and SACQ Institutional Attachment was 3.61% (*r*_sp_ = 0.10), whereas the unique association with self-efficacy was 3.24% (*r*_sp_ = 0.18).

#### 3.2.5. Predicting CAQ College Adaptation

The fifth simultaneous multiple regression analysis was conducted in order to assess the significant predictors of general university adjustment, measured by the CAQ scale. The results of this regression are described in Table 8. Taking as a whole, the set of independent variables made a significant contribution to the CAQ, *F* (10, 193) = 18.64, *p* < 0.001, *R*^2^= 0.49, _Adj_*R*^2^ = 0.47, and CISS Task, β = 0.32, *t* (193) = 4.46, *p* < 0.001, CISS Emotion, β = −0.37, *t* (193) = 0.7, *p* < 0.001, self-efficacy, β = 0.29, *t* (193) = 3.21, *p* < 0.01, relational availability, β = 0.20, *t* (193) = 3.21, *p* < 0.01, and self-esteem, β = −0.20, *t* (193) = −1.99, *p* < 0.05. The squared semi partial correlation coefficients indicated that the unique association between CISS Task and CAQ was 5.3% (*r*_sp_ = 0.23), between CISS Emotion and *CAQ* was 6.8% (*r*_sp_ = −0.26), between self-efficacy and CAQ was 2.3% (*r*_sp_ = 0.17), between relational availability and *CAQ* was 2.3% (*r*_sp_ = 0.17), and between self-esteem and CAQ was 1% (*r*_sp_ = −0.10).

## 4. Discussion

The transition from high school to university represents a life phase characterized by various personal, social, and academic shifts. All of these changes require adaptation, and they may be particularly stressful for students who are asked to regulate themselves to handle the demands of the new academic and social environment [7,8].

The primary aim of the current study was to examine university adjustment among first-year undergraduate students and investigate the possible predictors. Regarding the possible associations with individual variables, in line with previous research [4,12,19,24,28,31,32] we expected that coping styles, self-esteem, self-efficacy, and personality traits were significant predictors of university adjustment.

We found that scores on both CAQ and SACQ were quite high, meaning that our study sample did not find particular difficulties in facing the new social and academic demands. In addition, these satisfying levels of students’ university adjustment reflect features and qualities of the specific institution of the present study, with regard to teaching, social networks with teachers and colleagues, the quality or the amount of study, the curricular and extra-curricular activities, and the organization as a whole. Sure enough, some of the CAQ and SACQ items refer to the specific Department students were attending (e.g., *“I’m pleased now about my decision to attend this college in particular”, “Lately I have been giving a lot of thought to transferring to another college”, “I am very satisfied with the professors I have now in my courses”*).

Among the three different coping styles, our results showed that both problem- and emotion-focused coping styles reported significant relationships of the hypothesized direction, whereas avoidance-focused coping strategies did not predict either global levels of university adjustment or any of its specific subdimensions. A viable explanation of these unexpected results can be seen in the tricky conceptualization of this coping style and in the failure to discriminate escape from avoidance coping patterns. As Haskell et al. [41] recently argued, literature on coping problematically combines avoidance and escape behaviors into a single category. Avoidance consists in individuals’ anticipation of a negative or stressful situation, minimizing the likelihood of experiencing a potentially stressful stimulus, whereas escape involves the attempt of removing oneself from a stressor already experienced. From this perspective, the CISS items belonging to the Avoidance subscale seem to better reflect escape, rather than avoidance, as respondents were asked to indicate at what extent they performed the described behaviors while experiencing a stressful event. In addition, these authors also emphasized that neither avoidance nor escape behaviors can be seen as maladaptive coping styles, as although neither of them solve problems or stressors, they are useful to reduce their effect on individual’s wellbeing. Further, the CISS Avoidance subscale does not allow us to distinguish cognitive avoidance from behavioral avoidance, thus increasing the ambiguity and complexity of this coping style.

Surprisingly, self-esteem did not report any statistical predictions on any facets of university adjustment as measured by the SACQ, and it negatively affected the outcome variable as assessed by the CAQ. These findings can be read in the light of some empirical studies which emphasize the dark and sinister side of self-esteem [21,42]. According to these authors, individuals with high levels of self-esteem may tend to react badly to ego-threats, showing poor self-regulation and maladaptive behaviors. This prospective clearly contradicts the well supported view stating that self-esteem is a relevant factor in promoting several positive outcomes [22,23,24], but it reflects the complexity and heterogeneity of self-esteem’s conceptualization and operationalization. Moreover, self-esteem, measured by the Rosenberg SES, may also be affected by confounding self-representation biases, that is, by the individuals’ tendency to hinder or not disclose their own negative traits and feelings (social desirability). People with this type of high self-esteem (high self-esteem and high self-representation biases) have shown a greater level of sensitivity to stressful situations, acting defensively to avoid negative outcomes [21]. Nevertheless, we have no sufficient information to assert whether self-worth evaluations were distorted by social desirability, thus not allowing to discriminate overt from covert self-esteem.

In line with our expectations, self-efficacy was found to enhance both university adjustment as a whole (as assessed by the CAQ) and its facets (as measured by the SACQ). Interestingly, self-efficacy did not report any statistical effects on SACQ Psychological Adjustment subscale. As a possible interpretation, we can mention the dual model of mental health proposed by some authors [43,44,45], according to which psychological functioning is composed of two separate, yet correlated, dimensions: positive and negative mental health. A recent study [46] demonstrated that different mechanisms exist for the two dimensions of mental health in the context of stress-buffering process. From this point of view, the SACQ Psychological Adjustment subscale is composed of items describing both positive (wellness, good health) and negative (i.e., anxiety, mood disturbances) mental health, without discriminating one dimension from the other. This may likely have affected the lack of significant associations.

Finally, our findings showed that only relational availability and mental flexibility reported significant associations. The former was found to predict both global university adjustment, and some of its specific domains (social adjustment and institutional attachment); the latter reported a significant contribution to social domain only. Although both relational availability and mental flexibility reported associations with university adjustment of the hypothesized direction, they did not predict all the facets of the outcome variable, and the lack of relationships with the other personality traits let us claim that personality variables are less meaningful in predicting adaptation to college. From this perspective, our results are in line with previous studies [33] suggesting that the Five-Factors model conceptualizes personality traits too widely, minimizing their predictive role in adjustment to college.

As a strength, this study presents associations with coping strategies, self-efficacy, self-esteem, and personality traits in a unique study sample, adding a contribution to the extended international literature. This represents the added value of the current research, as no existing studies were found in which the influence of the considered variables on university adjustment has been jointly examined.

Nevertheless, there are several shortcomings to the study which limit the generalizations of our results. Firstly, the sample was lightly weighted towards women. Secondly, a high percentage of individuals were Psychology students (43.6% of the total sample), whereas other courses were underrepresented or even not represented. This can be considered as a threat for the representativeness of our sample, and did not allow us to investigate whether levels of adjustment to college varied according to the kind of studies. Indeed, the way students adapt to university life may be affected by the specific courses attended, the professors’ characteristics, and the quality of teaching. From this point of view, it should be beneficial that future research will consider an equal number of students enrolled in the diverse departments in order to examine whether differences in adjustment to college exist among these subgroups. 

Moreover, as we did not consider further participants’ characteristics (e.g., academic performance, socio-economic status, and physical and mental health) which may be associated with the way students adapt to university life, we suggest that, for a better interpretation of the results, additional studies should take into account this set of variables regarding participants’ information. This underlines that the study design has several potential sources of biases related to possible confounding variables which weaken the internal validity of the research. Not including further theoretically variables associated with university adjustment does not guarantee the correctness of our conclusions. 

In addition, the sample’s recruitment may represent a source of bias of the findings: the non-probabilistic procedure may further undermine the representativeness of our sample, and the informal contacts during which data were gathered did not guarantee uniformity and standardized conditions for data collection. Additional research using probabilistic sampling methods is needed to generalize results to larger populations, and standardized conditions for data collection are highly recommended to minimize results biases.

Furthermore, as this study was conducted at the end of the first academic year, students may have already adapted to university more than if they were in their first weeks of university. It may be helpful to assess the observed variables at the beginning of the year (around 4–6 weeks), in order to develop a broader understanding of the transition to university life. Lastly, the cross-sectional nature of the study did not allow us to make inferences about cause-and-effect associations. Longitudinal studies are required to explore the temporal relationships among the measured variables and to identify the possible predictors of the outcomes. Our findings have practical implications, suggesting the promotion and strengthening of students’ positive resources to increase the likelihood of a better adaptation to the transition to university life. For instance, it should be advantageous and useful that universities offer courses for freshmen during their first semester, held by professors but also by older students, with a focus on how to handle the academic demands and the challenges that it is possible to encounter in the new environment. In addition, universities should promote counseling services for students with difficulties in adjusting to university life in order to foster their personal skills in facing with university stressors. Additionally, the organization of support groups may create opportunities for sharing both positive and negative emotions related to the transition to university life, generating constructive moments, and developing a sense of shared experience. Promoting adjustment to college is a key factor strictly related to academic performance, achievement, and success, reducing withdrawal, and behavioral and/or mental disengagement. Future research may reproduce a similar study in public universities and evaluate the role of further variables (e.g., choice overload when choosing university classes [47], self-actualization [48], and boredom [49]). In addition, it may be useful to utilize a prospective design in which self-ratings would be collected before students start studying at college, to allow inferences about causation: sure enough, results from concurrent designs could be ambiguous in that assessments of personality might be reactions to adjustment status rather than predictors of it [31].

## 5. Conclusions

Our study provides a further understanding of adjustment among first-year university students, adding to the existing literature a contribution to better identify protective factors to prevent negative outcomes during the transition to university life. Taking into account a large set of variables as possible predictors, our results showed that self-efficacy, task-oriented coping strategies, followed by relational availability and mental flexibility, were positively associated with university adjustment, whereas emotion-oriented coping strategies reported significant associations of the opposite direction. These findings indicate that students’ beliefs in their own abilities to control life events, and the way they approach them, play a crucial role in predicting how well they adapt to university life. The present study highlights the need and the importance of using a multidimensional approach to examine college students’ adjustment, in order to have a broader and deeper comprehension of the investigated phenomenon.

## Figures and Tables

**Table 1:** Distribution of participants according to Faculty or Departments.

Faculty or Department	*N*
Psychology	89 (43.6%)
Sports Sciences	33 (16.2%)
Foreign Languages	27 (13.2%)
Economics	23 (11.3%)
Law	22 (10.8%)
Primary Education Sciences	6 (3%)
Architecture	3 (1.5%)
Engineering	1 (.5%)

**Table 2:** Descriptives for the observed variables.

	Min	Max	*M*	*Med*	*SD*	*S*	*K*
SACQ Academic Adjustment	89	212	155.76	157	24.59	−0.165	−0.401
SACQ Social Adjustment	71	169	128.18	129	20.30	−0.297	−0.499
SACQ Psychological Adjustment	44	133	91.76	93	18.75	−0.294	−0.572
SACQ Institutional Attachment	63	134	108.40	110	16.67	−0.709	0.040
CAQ	52	126	96.76	100	15.23	−0.367	−0.518
CISS Task	36	80	59.45	59	9.39	−0.041	−0.452
CISS Emotion	17	69	44.40	43	11.17	0.063	−0.457
CISS Avoidance	21	74	48.64	50	10.67	−0.125	−0.308
Self-efficacy	37	85	62.10	62	11.21	−0.049	−0.715
Activity	21	48	34.22	34	5.48	0.258	−0.516
Relational availability	21	47	33.90	34	4.57	0.011	−0.051
Diligence	16	48	34.41	34	5.60	0.021	0.073
Emotional stability	13	43	30.59	30	5.62	−0.227	0.185
Mental flexibility	22	47	32.60	32	3.91	−0.263	0.773
Self-esteem	16	48	32.47	32	6.40	0.050	0.038
Control	19	44	32.88	33	4.74	−0.080	0.113

Note. SACQ = Student Adaptation to College Questionnaire; CAQ = College Adaptation Questionnaire; CISS = Coping Inventory for Stressful Situations; *M* = Mean; *SD* = Standard Deviation; *Med.* = Median; *S* = Skewness; and *K* = Kurtosis.

**Table 3:** Correlations between the study variables.

Variable	1	2	3	4	5	6	7	8	9	10	11	12	13	14	15
1. SACQ Academic Adjustment	-														
2. SACQ Social Adjustment	0.732 **	-													
3. SACQ Psychological Adjustment	0.523 **	0.370 **	-												
4. Institutional Attachment	751 **	0.767 **	0.403 **	-											
5. CAQ	716 **	0.785 **	0.461 **	0.794 **	-										
6. CISS Task	0.521 **	0.446 **	0.141 *	0.447 **	0.471 *	-									
7. CISS Emotion	−0.415 **	−0.276 **	−0.583 **	−0.323 **	−0.413 *	−0.026	-								
8. CISS Avoidance	−0.051	0.053	−0.228 **	−0.056	−0.032	0.187 *	0.364 **	-							
9. Self-efficacy	0.606 **	0.582 **	0.302 **	0.555 **	0.590 **	0.556 **	−0.409 **	−0.042	-						
10. Activity	0.584 **	0.482 **	0.322 **	0.466 **	0.530 **	0.521 **	−0.446 **	−0.032	0.723 **	-					
11. Relational availability	0.321 **	0.400 **	−0.034	0.399 **	0.382 **	0.272 **	−0.005	0.148	0.386 **	0.350 **	-				
12. Diligence	0.572 **	0.418 **	0.333 **	0.437 **	0.432 **	0.516 **	−0.407 **	−0.097	0.623 **	0.712 **	0.280 **	-			
13. Emotional stability	0.326 **	0.315 **	0.350 **	0.254 **	0.371 **	0.288 **	−0.450 **	−0.054	0.483 **	0.518 **	0.206 **	0.356 **	-		
14. Mental flexibility	0.345 **	0.420 **	0.114	0.353 **	0.384 **	0.332 **	−0.227 **	0.054	0.462 **	0.505 **	0.455 **	0.344 **	0.480 **	-	
15. Self-esteem	0.450 **	0.393 **	0.367 **	0.355 **	0.439 **	0.430 **	−0.512 **	−0.021	0.683 **	0.651 **	0.236 **	0.447 **	0.735 **	0.539 **	-

Note. SACQ = Student Adaptation to College Questionnaire; CAQ = College Adaptation Questionnaire; and CISS = Coping Inventory for Stressful Situations. * *p* < 0.05, ** *p* < 0.01.

**Table 4:** Simultaneous Multiple Regression Analysis for variables predicting SACQ Social Adjustment.

Variable	Β	*r*_sp_	R^2^	_Adj_R^2^
CISS Task	0.20 **	0.15		
CISS Emotion	−0.19 **	−0.14		
CISS Avoidance	0.06	0.06		
Self-efficacy	0.39 ***	0.23		
Activity	0.01	0.01		
Relational availability	0.16 *	0.14		
Diligence	−0.03	0.02		
Emotional stability	0.02	0.01		
Mental flexibility	0.15 *	0.11		
Self-esteem	−0.19	−0.09		
Model			0.43	0.39

Note. * *p* < 0.05, ** *p* < 0.01, *** *p* < 0.001.

**Table 5:** Simultaneous multiple regression analysis for variables predicting SACQ Academic Adjustment.

Variable	Β	*r*_sp_	*R* ^2^	_Adj_ *R* ^2^
CISS Task	0.30 ***	0.22		
CISS Emotion	−0.30 ***	−0.21		
CISS Avoidance	0.00	0.00		
Self-efficacy	0.21 *	0.12		
Activity	0.10	0.06		
Relational availability	0.12	0.10		
Diligence	0.12	0.08		
Emotional stability	−0.06	−0.04		
Mental flexibility	0.01	0.01		
Self-esteem	−0.08	−0.04		
Model			0.52	0.49

Note. * *p* < 0.05, *** *p* < 0.001.

**Table 6:** Simultaneous multiple regression analysis for variables predicting SACQ Psychological Adjustment.

Variable	Β	*r*_sp_	*R* ^2^	_Adj_ *R* ^2^
CISS Task	0.11	0.08		
CISS Emotion	−0.50 ***	0.36		
CISS Avoidance	0.04	−0.03		
Self-efficacy	−0.01	−0.01		
Activity	−0.02	0.01		
Relational availability	−0.06	−0.05		
Diligence	0.09	0.06		
Emotional stability	0.11	0.08		
Mental flexibility	−0.09	−0.07		
Self-esteem	−0.02	−0.01		
Model			0.37	0.44

Note. *** *p* < 0.001.

**Table 7:** Simultaneous multiple regression analysis for variables predicting SACQ Institutional Attachment.

Variable	Β	*r*_sp_	*R* ^2^	_Adj_ *R* ^2^
CISS Task	0.28 ***	0.20		
CISS Emotion	−0.27 ***	−0.19		
CISS Avoidance	−0.04	−0.04		
Self-efficacy	0.31 ***	−0.18		
Activity	0.02	0.01		
Relational availability	0.23 ***	0.19		
Diligence	−0.01	−0.01		
Emotional stability	−0.06	−0.04		
Mental flexibility	−0.07	0.06		
Self-esteem	−0.16	−0.08		
Model			0.43	0.40

Note. *** *p* < 0.001.

**Table 8:** Simultaneous multiple regression analysis for variables predicting CAQ College Adaptation.

Variable	Β	*r*_sp_	*R* ^2^	_Adj_ *R* ^2^
CISS Task	0.32 ***	0.23		
CISS Emotion	−0.37 ***	−0.26		
CISS Avoidance	0.01	0.01		
Self-efficacy	0.29 **	0.17		
Activity	0.09	0.05		
Relational availability	0.20 **	0.17		
Diligence	−0.13	−0.08		
Emotional stability	0.05	0.04		
Mental flexibility	0.05	0.04		
Self-esteem	−0.20 *	−0.10		
Model			0.49	0.47

Note. * *p* < 0.05, ** *p* < 0.01, *** *p* < 0.001.

## Data Availability

The data used to support the findings of this study are available from the corresponding author upon request.

## References

[1] Santrock J.W. (2013). Life-Span Development.

[2] Maunder R.E. (2017). Students’ peer relationships and their contribution to university adjustment: The need to belong to in the university community. J. Furth. High. Educ..

[3] Rahat E., Ilhan T. (2016). Coping styles, social support, relational self-construal, and resilience in predicting students’ adjustment to university life. Educ. Sci. Theory Pract..

[4] Credé M., Niehorster S. (2012). Adjustment to college as measured by the Student Adaptation to College Questionnaire: A quantitative review of its structure and relationships with correlates and consequences. Educ. Psychol. Rev..

[5] Duchesne S., Ratelle C.F., Larose S., Guay F. (2007). Adjustment trajectories in college science programs: Perceptions of qualities of parents’ and college teachers’ relationships. J. Couns. Psychol..

[6] Pittman L.D., Richmond A. (2008). University belonging, friendship quality, and psychological adjustment during the transition to college. J. Exp. Educ..

[7] Ababu G.B., Yigzaw A.B., Besene Y.D., Alemu W.G. (2018). Prevalence of adjustment problem and its predictors among first-year undergraduate students in Ethopian University: A cross-sectional institution based study. Psychiatry J..

[8] Beiter R., Nash R., McCrady M., Rhoades D., Linscomb M., Clarahan M., Sammut S. (2015). The prevalence and correlates of depression, anxiety, and stress in a sample of college students. J. Affect. Disord..

[9] Raza S.A., Qazi W., Yousufi S.Q. (2020). The influence of psychological, motivational, and behavioral factors on university students’ achievement: The mediating effect of academic adjustment. J. Appl. Res. High. Educ..

[10] van Rooij E.C.M., Jansen E.P.W.A., van de Grift W.J.C.M. (2018). First-year university academic success: The importance of academic adjustment. Eur. J. Psychol. Educ..

[11] English T., Davis J., Wei M., Gross J.J. (2017). Homesickness and adjustment across the first year of college: A longitudinal study. Emotion..

[12] Quan L., Zhen R., Yao B., Zhou X. (2014). The effects of loneliness and coping style on academic adjustment among college freshmen. Soc. Behav. Pers. Int. J..

[13] Baker R.W., Siryk B. (1984). Measuring adjustment to college. J. Couns. Psychol..

[14] Lazarus R.S., Folkman S. (1984). Stress, Appraisal and Coping.

[15] Park C.L., Adler N.E. (2003). Coping style as a predictor of health and well-being across the first year of medical school. Health Psychol..

[16] Ben-Zur H. (2009). Coping styles and affect. Int. J. Stress Manag..

[17] Cousins C., Servaty-Seib H.L., Lockman J. (2017). College student adjustment and coping: Bereaved and nonbereaved students. OMEGA-J. Death Dying.

[18] Katz S., Somers C.L. (2015). Individual and Environmental Predictors of College Adjustment: Prevention and Intervention. Curr. Psychol..

[19] Sevinc S., Gizir C.A. (2014). Factors negatively affecting university adjustment from the views of first-year university students: The case of Mersin University. Educ. Sci. Theory Pract..

[20] Rosenberg M. (1979). Conceiving the Self.

[21] Lambird K.H., Mann T. (2006). When do ego threats lead to self-regulations failure? Negative consequences of defensive high self-esteem. Pers. Soc. Psychol. Bull..

[22] Afolabi O.A. (2014). Do self-esteem and family relations predict prosocial behavior and social adjustment of fresh students?. High. Educ..

[23] Hernandez R.M.R. (2017). Freshmen students’ self-esteem and adjustment to college in higher education institution in Calapan city, Philipines. Asia Pac. Multidiscip. Res..

[24] Nordstrom A.H., Goguen L.M.S., Hiester M. (2014). The Effect of Social Anxiety and Self-Esteem on College Adjustment, Academics, and Retention. J. Coll. Couns..

[25] Pasha H.S., Munaf S. (2013). Relationship of self-esteem and adjustment in traditional university students. Procedia-Soc. Behav. Sci..

[26] Bandura A. (1997). Self-Efficacy: The Exercise of Control.

[27] Brady-Amoon P., Fuertes J.N. (2011). Self-efficacy, self-rated abilities, adjustment, and academic performance. J. Couns. Dev..

[28] Girelli L., Alivernini F., Lucidi F., Cozzolino M., Savarese G., Sibilio M., Salvatore S. (2018). Autonomy supportive contexts, autonomous motivation, and self-efficacy predict academic adjustment of first-year university students. Front. Educ..

[29] Bardi A., Riff C.D. (2007). Interactive effects of traits on adjustment to a life transition. J. Pers..

[30] Klimstra T.A., Noftle E.E., Luyckx K., Goossens L., Robins R.W. (2018). Personality development and adjustment in college: A multifaceted, cross-national view. J. Pers. Soc. Psychol..

[31] Kurtz J.E., Puher M.A., Cross N.A. (2012). Prospective Prediction of College Adjustment Using Self- and Informant-Rated Personality Traits. J. Pers. Assess..

[32] Martin A.J., Nejad H.G., Colmar S., Liem G.A.D. (2013). Adaptability: How Students’ Responses to Uncertainty and Novelty Predict Their Academic and Non-Academic Outcomes. J. Educ. Psychol..

[33] Lidy K.M., Kahn J.H. (2006). Personality as a Predictor of First-Semester Adjustment to College: The Mediational Role of Perceived Social Support. J. Coll. Couns..

[34] Faul F., Erdferlder E., Lang A.-G., Buchner A. (2007). G*Power 3: A flexible statistical power analysis program for the social, behavioral, and biomedical sciences. Behav. Res. Methods.

[35] Baker R.W., Siryk B. (1989). SACQ: Student Adaptation to College Questionnaire Manual.

[36] Crombag H.F.M. (1968). Studiemotivatie en Studieattitude [Study Motivation and Study Attitude].

[37] Pierro A. (1997). Caratteristiche strutturali della scala di General Self-Efficacy. Giunti Organ. Spec..

[38] Endler N., Parker J.D.A. (1990). Coping Inventory for Stressful Situations (CISS): Manual.

[39] Sirigatti S., Steganile C. (2009). CISS-Coping Inventory for Stressful Situation. Standardizzazione e Validazione Italiana.

[40] Mancinelli M.R. (1998). QA—Questionario di Autovalutazione.

[41] Haskell A.M., Britton P.C., Servatius R.J. (2019). Toward an assessment of escape/avoidance coping in depression. Behav. Brain Res..

[42] Jordan C.H., Spencer S.J., Zanna M.P., Hoshino-Browne E., Correll J. (2003). Secure and defensive high self-esteem. J. Pers. Soc. Psychol..

[43] Suldo S.M., Shaffer E.J. (2008). Looking beyond psychopathology: The dual-factor model of mental health in youth. Sch. Psychol. Rev..

[44] Wang X., Zhang D., Wang J. (2011). Dual-factor model of mental health: Surpass the traditional mental health model. Psychology.

[45] Weich S., Brugha T., King M., McManus S., Bebbington P., Jenkins R., Cooper C., McBride O., Stewart-Brown S. (2011). Mental well-being and mental illness: Findings from the Adult Psychiatric Morbidity Survey for England 2007. Br. J. Psychiatry.

[46] Schönfeld P., Brailovskaia J., Bieda A., Zhang X.C., Margraf J. (2016). The effects of daily stress on positive and negative mental health: Mediation through self-efficacy. Int. J. Clin. Heal. Psychol..

[47] Misuraca R., Teuscher U., Faraci P. (2016). Is more choice always worse? Age differences in the overchoice effect. J. Cogn. Psychol..

[48] Faraci P., Cannistraci S. (2015). The Short Index of Self-Actualization: A factor analysis study in an Italian sample. Int. J. Psychol. Res..

[49] Craparo G., Faraci P., Gori A., Hunter J.A., Hunter A., Pileggi V., Costanzo G., Lazzaro A., Eastwood J.D. (2017). Validation of the Italian version of the Multidimensional State Boredom Scale (MSBS). Clin. Neuropsychiatry.

